# Aspirin inhibits adipogenesis of tendon stem cells and lipids accumulation in rat injury tendon through regulating PTEN/PI3K/AKT signalling

**DOI:** 10.1111/jcmm.14622

**Published:** 2019-09-26

**Authors:** Yunjiao Wang, Gang He, Feng Wang, Chenke Zhang, Zilu Ge, Xiaolong Zheng, Honghao Deng, Chengsong Yuan, Binghua Zhou, Xu Tao, Jiqiang Zhang, Kanglai Tang

**Affiliations:** ^1^ Department of Orthopaedics/Sports Medicine Center, State Key Laboratory of Trauma, Burn and Combined Injury Southwest Hospital, Third Military Medical University Chongqing China; ^2^ Department of Neurology Third Military Medical University Chongqing China

**Keywords:** adipogenesis, aspirin, tendinopathy, tendon stem cells

## Abstract

Tendon injury repairs are big challenges in sports medicine, and fatty infiltration after tendon injury is very common and hampers tendon injury healing process. Tendon stem cells (TSCs), as precursors of tendon cells, have shown promising effect on injury tendon repair for their tenogenesis and tendon extracellular matrix formation. Adipocytes and lipids accumulation is a landmark event in pathological process of tendon injury, and this may induce tendon rupture in clinical practice. Based on this, it is important to inhibit TSCs adipogenesis and lipids infiltration to restore structure and function of injury tendon. Aspirin, as the representative of non‐steroidal anti‐inflammatory drugs (NSAIDs), has been widely used in tendon injury for its anti‐inflammatory and analgesic actions, but effect of aspirin on TSCs adipogenesis and fatty infiltration is still unclear. Under adipogenesis conditions, TSCs were treated with concentration gradient of aspirin. Oil red O staining was performed to observe changes of lipids accumulation. Next, we used RNA sequencing to compare profile changes of gene expression between induction group and aspirin‐treated group. Then, we verified the effect of filtrated signalling on TSCs adipogenesis. At last, we established rat tendon injury model and compared changes of biomechanical properties after aspirin treatment. The results showed that aspirin decreased lipids accumulation in injury tendon and inhibited TSCs adipogenesis. RNA sequencing filtrated PTEN/PI3K/AKT signalling as our target. After adding the signalling activators of VO‐Ohpic and IGF‐1, inhibited adipogenesis of TSCs was reversed. Still, aspirin promoted maximum loading, ultimate stress and breaking elongation of injury tendon. In conclusion, by down‐regulating PTEN/PI3K/AKT signalling, aspirin inhibited adipogenesis of TSCs and fatty infiltration in injury tendon, promoted biomechanical properties and decreased rupture risk of injury tendon. All these provided new therapeutic potential and medicine evidence of aspirin in treating tendon injury and tendinopathy.

## 1 INTRODUCTION

Tendons are anatomical structures connecting muscles to bone which generate transmission of forces, thereby ensuring joint movements. Tendon injuries have become a common clinical disorder due to overuse or age‐related degeneration.^1^ Injury tendons heal slowly and hardly restore the structure integrity and mechanical strength of intact tendon, which often results in clinical and patients' burden.^2^

TSCs have been reported for self‐renewal ability, colony formation ability, multi‐differentiation potential, which make them differentiate into adipocytes, tenocytes and osteocytes.^3^, ^4^, ^5^ Adipocytes and lipids accumulation is a remarkable pathological process in tendon injury, and this may induce risk of tendon rupture in clinical practice. So to inhibit TSCs adipogenic differentiation is vital to regain tendon structure and function.

The treatment for tendon injury includes NSAIDs oral administration, corticosteroids local injection, extracorporeal shock wave therapy, gene therapy and tissue engineering therapy.^6^, ^7^, ^8^ Among that, NSAIDs have been widely used in clinical practice for their anti‐inflammation and relieving pain through inhibiting prostaglandin. Aspirin, as the classic representative of NSAIDs, has been used in many clinical fields, such as cardiovascular system,^9^ central nervous system,^10^ some cancers^11^ and tendinopathy/tendon injury. Its anti‐inflammation and pain relief effect have been widely accepted, but evidence of its effect on TSCs differentiation is lacking, especially its effect on adipogenic differentiation and injury tendon healing.

The present study aimed to investigate the effect of aspirin on TSCs adipogenic differentiation and fatty infiltration in injury tendon, find out the molecular difference and related signalling through RNA sequencing and provide new therapeutic potential and medicine evidence of aspirin in treating tendon injury and tendinopathy.

## 2 MATERIALS AND METHODS

### 2.1 Ethics statement

All animals were treated according to institutional guidelines for laboratory animal treatment and care. All experimental procedures were approved by the Animal Research Ethics Committee of Third Military Medical University, China.

### 2.2 Animal model establishment

We followed the methods as the previous studies.^12^, ^13^, ^14^, ^15^ Twenty‐four male Sprague‐Dawley rats were randomly divided into three groups: control group, injury model group and aspirin treatment group. Firstly, 30 μl collagenase I (10 mg/ml) were injected into both Achilles tendons of rats in injury group and aspirin treatment group to establish tendinopathy model. One week after collagenase I injection, aspirin (30 mg/d) was given to each rat in aspirin treatment group for 4 weeks.

### 2.3 Isolation and identification of rat TSCs

We performed the isolation and identification of rat TSCs as our previously studies.^12^, ^16^ Briefly, rats in all groups were euthanized, and then, Achilles tendons were carefully collected. The samples were cut into pieces in sterile phosphate buffer saline (PBS) and digested in collagenase I (3 mg/ml; Sigma‐Aldrich) for 2.5 hours at 37°C. A single‐cell suspension was made through cell strainer (Becton Dickinson). The released cells were washed in PBS and centrifuged at 300 *g* for 5 minutes, then incubated in Dulbecco's Modified Eagle's Medium (DMEM; Gibco) with 10% foetal bovine serum (Invitrogen). Isolated cells were cultured under conditions of 37°C with 5% CO_2_ for 2 days, then washed in PBS to discard non‐adherent cells. On day 7 of culture, Trypsin‐EDTA solution (Sigma‐Aldrich) was used to digest TSCs, and TSCs were mixed together and cultured as passage (P) 0 cells. Cells from P1‐P3 were used in the next studies.

### 2.4 Adipogenic differentiation of TSCs

Adipogenic induction medium (Cyagen) was prepared for inducing adipogenesis of TSCs. Throughout the experiments, aspirin (Sigma‐Aldrich) was dissolved in dimethyl sulfoxide (DMSO), and induction medium was changed every 3 days. Seeding density of TSCs onto the 6‐well plate was 6 × 10^4^ cells/well, and TSCs were treated with 0, 0.25, 0.5, 1 or 2 mM aspirin for 24 hours or with 2 mM aspirin for 3, 7 and 14 days. In inhibition analysis, inhibitors of PI3K/AKT signalling pathway VO‐Ohpic (MedChemExpress) and IGF‐1 (Beyotime Biotechnology) were added into TSCs with induction medium and aspirin treatment for 3 days.

### 2.5 RNA‐seq and data analysis

Total RNA was collected from TSCs treated with induction medium and induction medium with aspirin through TRIzol Reagent (Takara). Solexa pipeline v1.8 (Off‐Line Base Caller software, v1.8 Illumina) was used to perform image analysis and base calling. FastQC software (KangChen Bio‐tech) was used to assess total RNA samples. Reference genome using Hisat2 software was used to align the trimmed reads. StringTie was used to estimate the transcript abundances of each sample, R package Ballgown was used to calculate the FPKM value for differentially expressed genes and transcripts. StringTie and Ballgown were used to predict novel genes and transcripts, and CPAT was used to assess the coding potential of those sequences. rMATS.42 Principle Component Analysis was used to detect Alternative splicing events and plots, and gene expression level was used for correlation analysis. The differentially expressed genes in R, Python or shell environment for statistical computing and graphics were used to perform Hierarchical Clustering, Gene Ontology, scatter plots, volcano plots and pathway analysis.^17^

### 2.6 Protein extraction and Western blot

Protein extraction and Western blot analysis were performed as our previously study.^16^ After treatment, the cells were washed twice with ice‐cold PBS and lysed in lysis buffer containing a mixture of proteinase inhibitors (Thermo Fisher Scientific Inc). BCA protein assay kit (Thermo Fisher Scientific Inc) was used to measure total protein concentrations, and SDS polyacrylamide gel electrophoresis was performed after adding equal amounts of proteins samples (30 µg/lane) and then proteins were transferred onto polyvinylidene difluoride membranes. After that, the membranes were blocked with 5% non‐fat milk containing 0.1% TBSTween at room temperature for 2 hours, and then incubated with primary antibodies at 4°C overnight. We used the following primary antibodies: anti‐ap2 (Proteintech, 1:2000), anti‐C/EBPα (Cell Signaling Technology, 1:2000), anti‐PPARγ (Proteintech, 1:2000), anti‐PTEN (Bioss, 1:2000), anti‐PI3K (Proteintech, 1:2000), anti‐Phospho (P)‐PI3K (Proteintech, 1:2000), anti‐AKT (Bioss, 1:2000) and anti‐P‐AKT (Bioss, 1:2000). Glyceraldehyde 3‐phosphate dehydrogenase (GAPDH; Proteintech, 1:5000) was used as internal control. After incubation with primary antibodies, membranes were washed in 0.1% TBST for three times and incubated in goat anti‐rabbit secondary antibodies (Proteintech, 1:2000) for 2 hours at room temperature. Enhance chemiluminescence detection kit (GE Healthcare) was used to visualize and capture the protein images.

### 2.7 Real‐time quantitative PCR

The mRNA expression levels of adipogenesis related gene were determined by qRT‐PCR. TRIzol regent was used to extract total RNA from cells, according to the protocol provided by the manufacturer (Takara). Superscript III first‐strand synthesis kit (TaKaRa) was used to synthesize cDNA from total RNA. SYBR Green RT‐PCR kit (TaKaRa) and an ABI Prism 7900 Sequence Detection System (PE Applied Biosystems) were used to perform qPCR. Expression of the housekeeping gene GAPDH was used as relative expression control.

### 2.8 Immunostaining

Achilles' tendons were cut coronally to prepare serial frozen sections (5 μm) as previously study.^16^, ^18^ Briefly, sections were rewarmed at room temperature for 30 minutes and washed thrice in PBS every 5 minutes. 0.1% Triton 100 and 5% BSA were used to punch and block the samples for 1 hour, and sections were incubated with adipogenic differentiation markers ap2 (Abcam, 1:200) and PPARγ (Abcam, 1:200) at 4°C overnight. After that, sections were incubated with goat anti‐rabbit IgG H&L (Dylight‐594; Proteintech, 1:200) for 2 hours in the darkroom. After washed 5 minutes for four times, sections were counterstained with DAPI for 5‐8 minutes, and then, fluorescence quencher was used for the sections.

### 2.9 Biomechanical testing

We performed the biomechanical test as previous study.^19^ Briefly, the Achilles' tendons with upper and lower bony ends were firstly isolated. The two bony ends of Achilles' tendon were fixed on a custom‐made testing jig with two clamps. The calcaneus end was fixed on the lower clamp site while the tibia end was fixed on the upper clamp site. The mechanical testing machine was then connected to testing system of computer. After the operating parameters were entered, the biomechanical testing started on. Six to eight samples were used for each group.

### 2.10 Statistical analysis

Mean ± standard deviation (SD) was used to express all values. Comparison between two groups was calculated by the Student's *t* test. Multiple comparisons were calculated by one‐way analysis of variance followed by Fisher's tests. *P* < .05 was considered to be significantly different.

## 3 RESULTS

### 3.1 Aspirin inhibits adipogenic differentiation of TSCs

**Figure 1:**
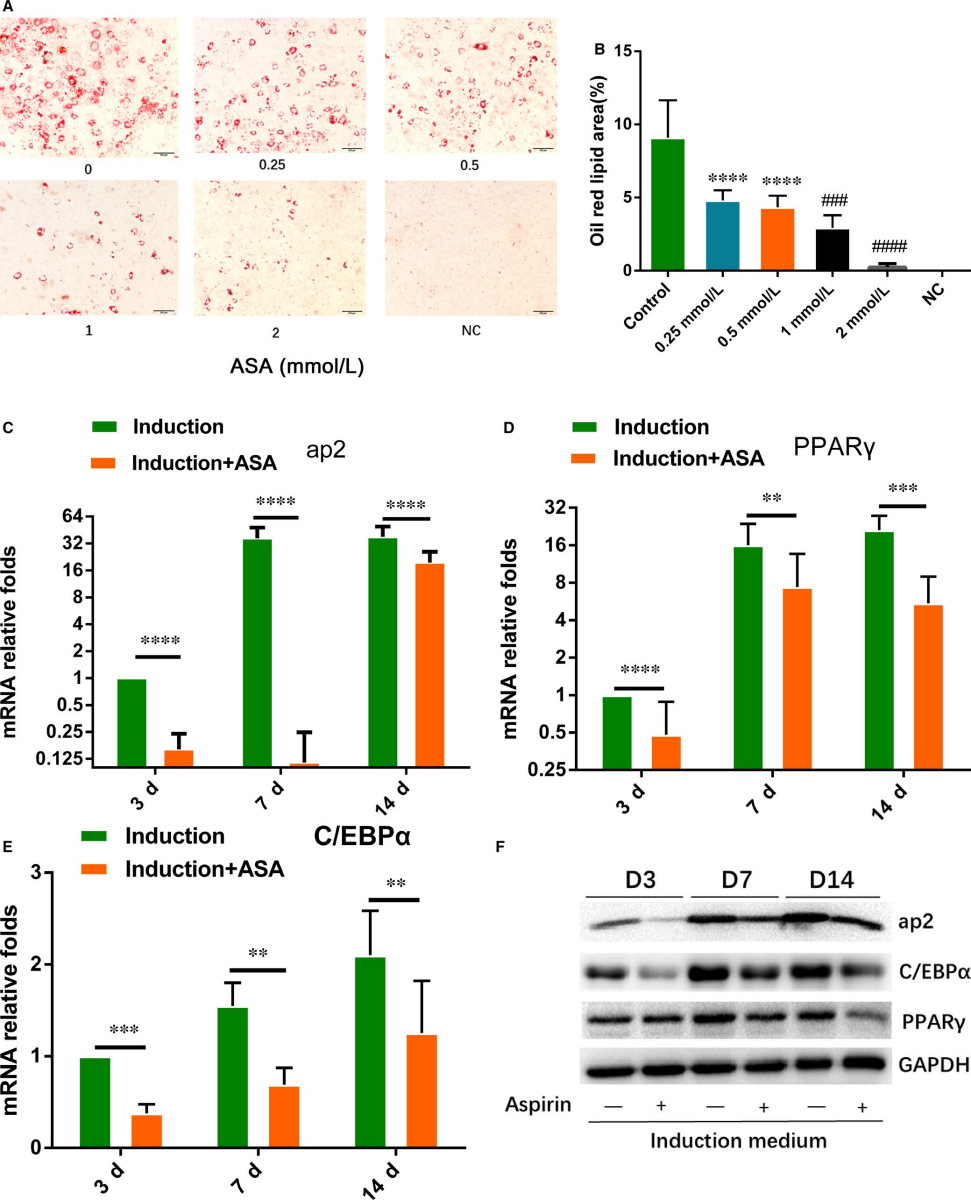
Effects of aspirin on the adipogenic differentiation of TSCs. A, TSCs were treated with increasing concentration (0‐2 mM) of aspirin under adipogenic induction medium for 14 d, and the Oil Red O staining was performed, Scale bars, 100 μm. B, Percentage of oil red lipid area was measured. C‐E, The relative expression levels of ap2, PPARγ and C/EBPα in induction group and induction with aspirin group at 3, 7 and 14 d were evaluated by qRT‐PCR. GAPDH was used as an internal control. F, Protein expression of ap2, PPARγ and C/EBPα in induction group and induction with aspirin group at 3, 7 and 14 d was evaluated by Western blot. GAPDH was used as an internal. *P < .05, **P < .01, ***P < .001, ****P < .000, #: vs. 0.5mM group, N = 3

To testify effect of aspirin on TSCs adipogenic differentiation, TSCs were treated by gradient concentration of aspirin with adipogenic induction medium. Oil red O staining showed that aspirin significantly inhibited lipid droplets formation with increasing concentration of aspirin (Figure 1A,B). qRT‐PCR showed that aspirin significantly lowered the gene expression of *ap2*, *PPARγ* and *C/EBPα* at 3, 7 and 14 days (Figure 1C‐E). Western blot also showed that protein expression of ap2, PPARγ and C/EBPα was inhibited by aspirin at 3, 7 and 14 days (Figure 1F).

### 3.2 RNA‐seq analysis of gene expression profile of ASA‐treated and untreated TSCs induced by adipogenic differentiation culture

**Figure 2:**
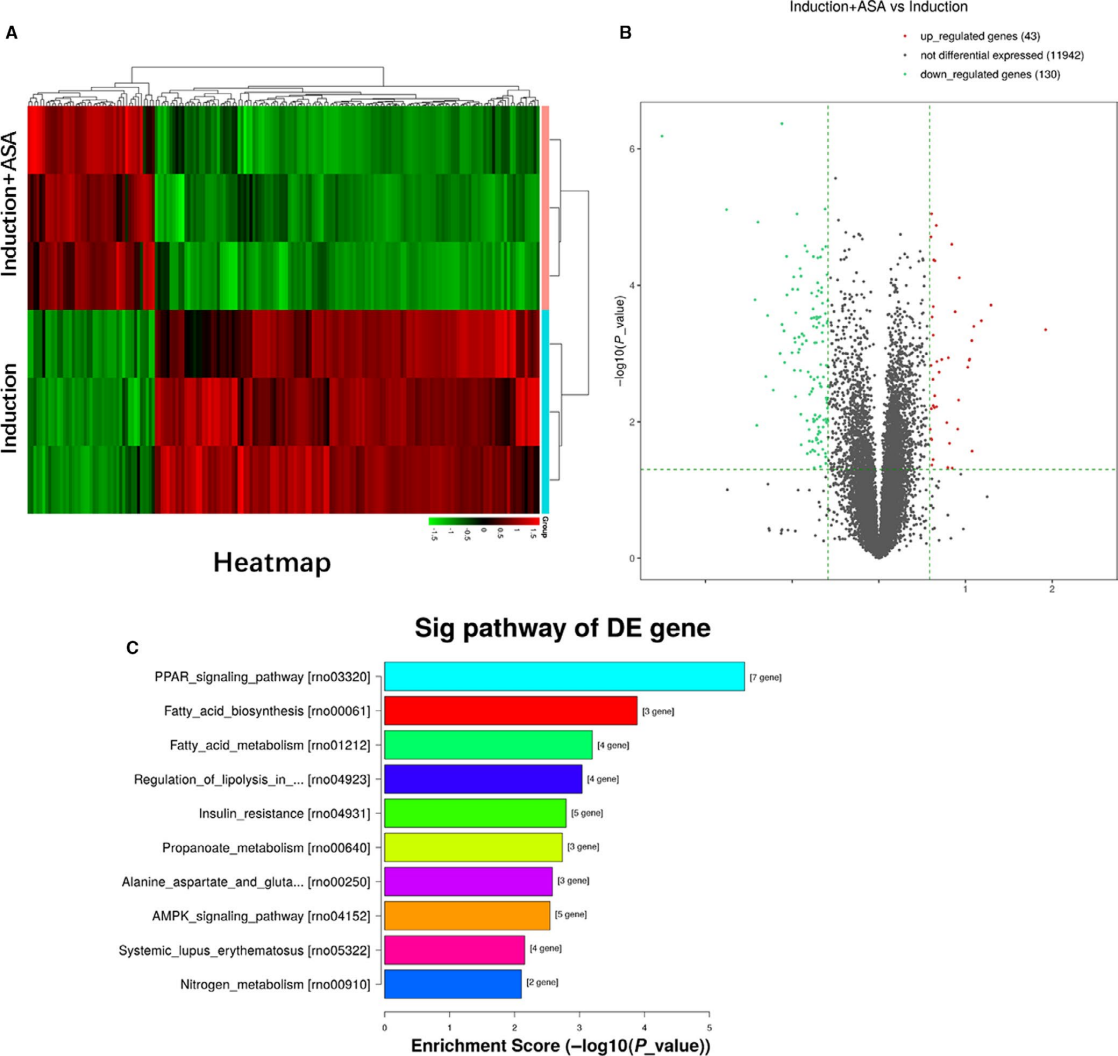
RNA‐seq analysis of gene expression profile of TSCs in the adipogenic induction group and induction with aspirin group. A, Heatmap depicting expression levels of genes between TSCs in induction group and that in induction with aspirin group. In total, 173 genes of TSCs were differentially expressed between induction group and induction with aspirin group. B, Volcano map of the differentially expressed genes of TSCs between induction group and induction with aspirin group. C, Top 10 enriched signalling pathways analysed by the KEGG analysis

In order to analyse the underlying mechanism related to the changes of adipogenic differentiation between aspirin treatment group and induction group, RNA sequencing was used to observe changes of gene expression profiles of TSCs. From results of the heatmap and volcano map (Figure 2A,B), 43 genes and 130 genes with log2 ratio above 2 were up‐ and down‐regulated, respectively, in the two groups. Kyoto Encyclopedia of Genes and Genomes (KEGG) showed that top ten down‐regulated signalling pathways in the two groups were enriched as shown (Figure 2C). The results showed that top four pathways, including PPARγ signalling, fatty acid biosynthesis, fatty metabolism and regulation of lipolysis, are all related to decreasing formation of fatty acids. The results above showed that fatty synthesis metabolism was inhibited in aspirin treatment group. From the results, we found that gene expression of *PTEN* was up‐regulated in aspirin treatment group, and PTEN was the upstream of PI3K/AKT signalling. So PTEN/PI3K/AKT signalling was enrolled into the next studies.

### 3.3 Aspirin inhibited adipogenesis of TSCs through inhibiting PTEN/PI3K/AKT signalling pathway

**Figure 3:**
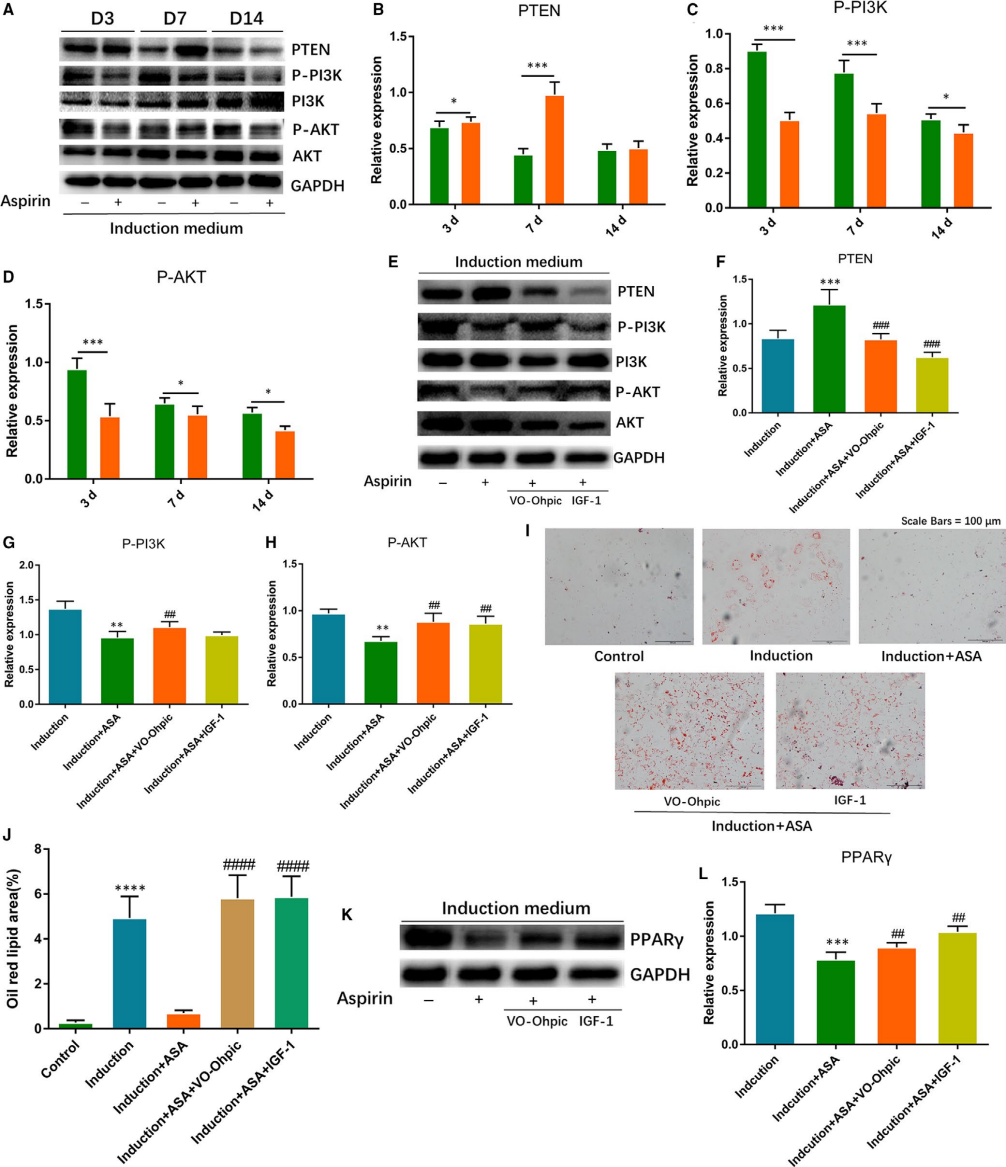
Aspirin inhibited adipogenic differentiation of TSCs through regulating PTEN/PI3K/AKT signalling. TSCs were treated with adipogenic induction medium and induction medium with aspirin for 3, 7 and 14 d. A‐D, Western blot analysis of protein expression level of PTEN, PI3K and AKT. GAPDH was used as an internal. E‐H, Under adipogenic induction medium with aspirin, we added VO‐Ohpic (2.5 μM) and IGF‐1 (10 ng/ml) into the TSCs for 3 d, comparing with induction group and induction with aspirin group. Western blotting showed the level of PTEN, PI3K and AKT. GAPDH was used as an internal. I‐J, Oil red staining showed fatty lipids accumulation changes among the five groups. K‐L, Western blotting showed the level changes of PPARγ among the five groups. GAPDH was used as an internal. *P < .05, **P < .01, ***P < .001, ****P < .000, *: vs. Control or Induction group, #: vs. Induction + ASA group, N = 3

To verify the effect of aspirin on PTEN/PI3K/AKT pathway, we firstly observed expression changes of PTEN, P‐PI3K and P‐AKT through Western blot. According to the results, we found that expression of P‐PI3K and P‐AKT decreased significantly at 3, 7 and 14 days. PTEN, which was the upstream inhibitor of PI3K/AKT pathway, was significantly elevated at 3 and 7 days, and was slightly up‐regulated at 14 days (Figure 3A‐D). To investigate if PTEN/PI3K/AKT signalling was engaged in aspirin‐inhibited adipogenesis, we added the activators of the signalling, VO‐Ohpic and IGF‐1, into TSCs before adding aspirin. Firstly, we verified the activation effect of VO‐Ohpic and IGF‐1 on the signalling. Western blotting showed that VO‐Ohpic and IGF‐1 significantly decreased PTEN expression and elevated the levels of P‐PI3K and P‐AKT (Figure 3E‐H). After that, we explored that if the inhibition of adipogenesis was reversed after two activators treatment. The results showed that the level of the adipogenesis effect in the group with activators was significantly higher than that of aspirin group. The two activators reversed the aspirin‐inhibited fatty droplets formation and related markers of PPARγ (Figure 3I‐L).

### 3.4 Aspirin inhibits lipids formation in injury tendon

**Figure 4:**
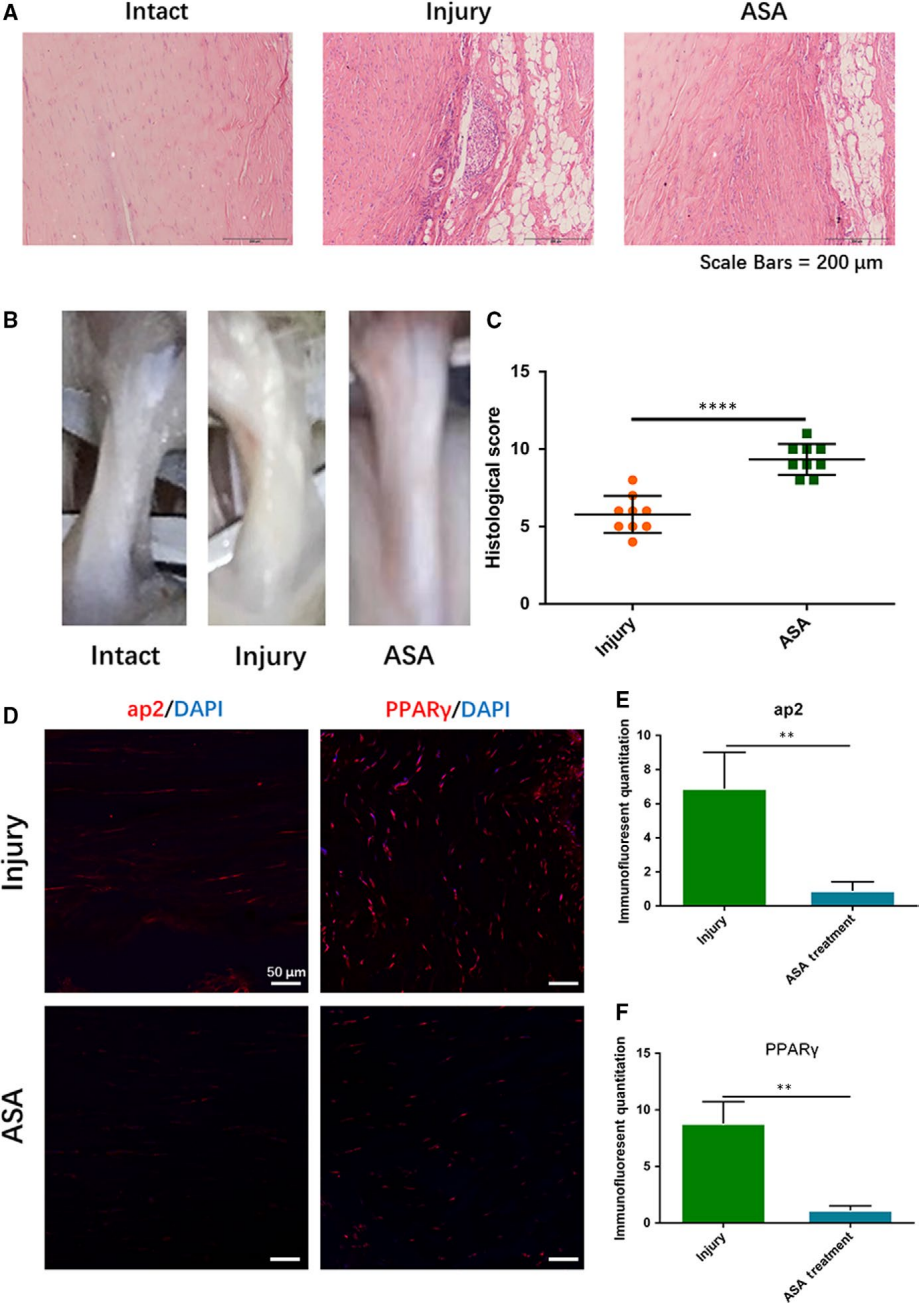
Effect of aspirin on adipogenic healing. A, Intact tendon, injury tendon and ASA treatment tendon observed through H&E staining. B, Gross appearance of three group tendons. C, Histological score of injury tendon and ASA treatment tendon, N = 9. D‐F, Immunostaining showed the level of ap2 and PPARγ between injury tendon and ASA treatment tendon. **P < .01, ****P < .000, N = 3

Four weeks after ASA treatment, tendon samples were harvested for tendon healing analysis and adipogenic observation. H&E staining showed that fatty lipids in aspirin treatment group significantly decreased compared with injury group (Figure 4A). General observation showed that less fatty infiltration occurred in injury tendon after aspirin treatment (Figure 4B), and histological score in aspirin‐treated group was significantly higher than that in injury group (Figure 4B,C). Immunostaining results showed that the levels of ap2 and PPARγ were significantly down‐regulated after aspirin treatment (Figure 4D‐F).

### 3.5 Aspirin increased the biomechanical properties of the injury tendon

**Figure 5:**
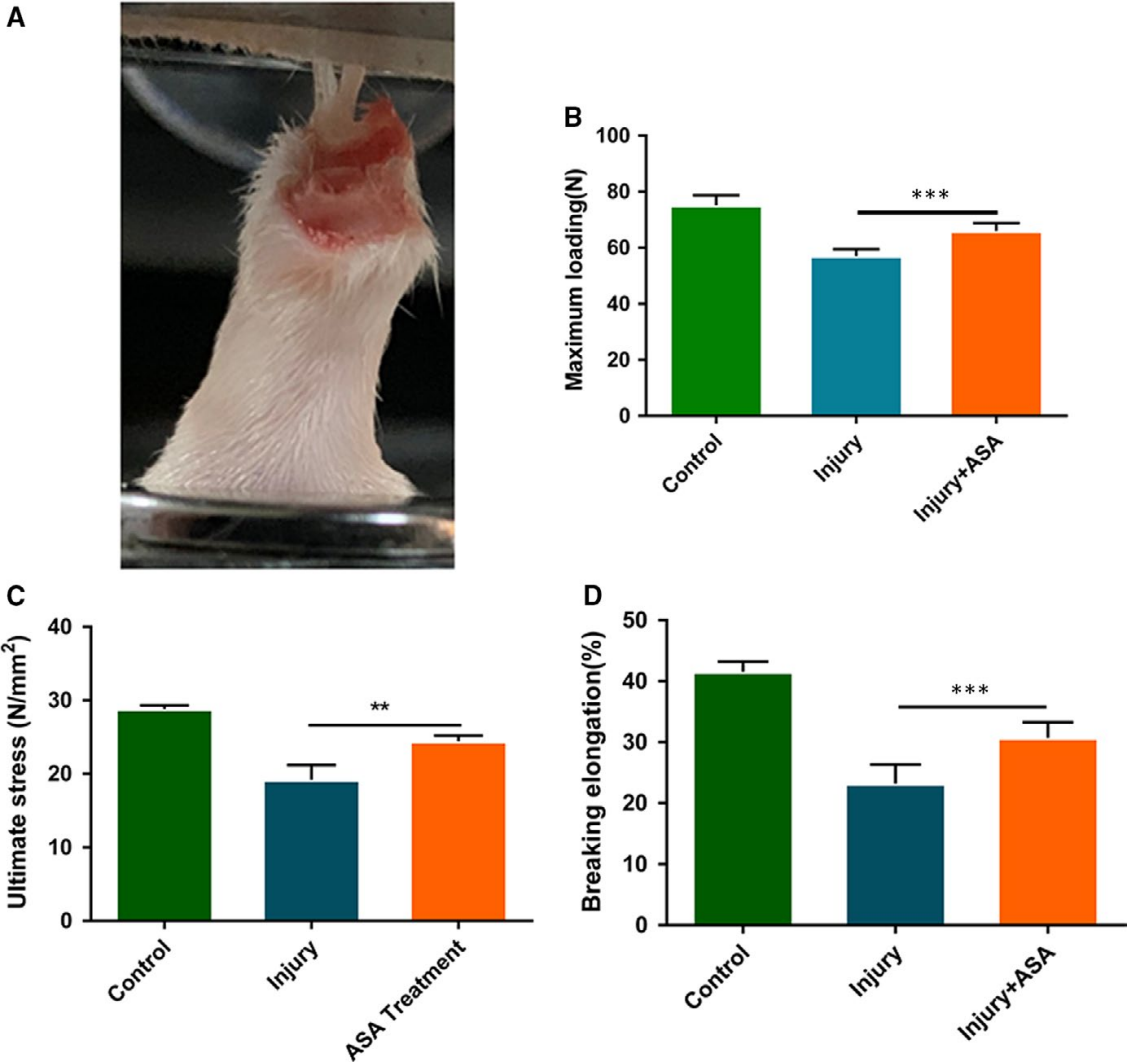
Effect of aspirin on biomechanical characters in injury tendon. A, Biomechanical test was performed. B‐D, Biomechanical properties including maximum loading, ultimate stress and breaking elongation were measured among three groups. **P < .01, ***P < .001, N = 6‐8

Four weeks after aspirin treatment, tendon samples were harvested for biomechanical test (Figure 5A). Biomechanical test showed that maximum loading after aspirin treatment was significantly elevated compared with injury tendons (Figure 5B). Ultimate stress and breaking elongation in aspirin treatment group were also significantly increased compared to the injury group (Figure 5C,D). The results showed that aspirin treatment decreased rupture risk of injury tendon.

## 4 DISCUSSION

Fatty infiltration and metaplasia of adipose tissue is very common after tendon injury. Some factors induce TSCs differentiate into adipocytes and generate fatty lipids. How to inhibit and reverse the fatty infiltration is an important issue for injury tendon healing process. Aspirin and its ramification, a representative of NSAIDs, have been widely used in clinical for their pain relief and anti‐inflammation effect through inhibiting the function of cyclooxygenase enzymes and prostaglandins,^20^ and TSCs play an irreplaceable role in repair process of tendinopathy, but the effect of aspirin on adipogenic differentiation of TSCs and fatty infiltration is still unclear. For the first time, the present study demonstrated that aspirin inhibited adipogenesis of TSCs through inhibition of PTEN/PI3K/AKT signalling pathway, decreased fatty infiltration and increased mechanical properties of injury tendon.

To make clear the effect of aspirin on adipogenic differentiation of TSCs, we firstly explored oil red staining and expression of fat formation related markers ap2, PPARγ and C/EBPα. The results revealed that aspirin inhibited the fat droplets and related markers formation. Some reports have demonstrated the effect of aspirin on cells differentiation. Yuan et al^21^ have proved that aspirin promoted the osteogenic potential and bone regeneration of human periodontal ligament stem cells (hPDLSCs) in vitro and in vivo. Also, Abd Rahman et al^22^ showed that low‐dose aspirin promoted cell growth and osteogenic differentiation of PDLSCs through up‐regulating the expression genes related to cell proliferation, tissue regeneration and differentiation. Zeng et al^23^also reported that aspirin inhibits osteoclastogenesis through inhibiting the activation of NF‐κB and MAPKs in RAW264.7 cells induced by RANKL. Up to now, there are still no related reports on effect of aspirin on adipogenic differentiation and fatty acids formation.

To make clear the underlying mechanism, we utilized the RNA sequencing to explore the molecular difference of gene expression between the two groups. The results showed that *PTEN*, which was upstream of PI3K/AKT signalling, was up‐regulated by aspirin. Based on this, we filtrated the PTEN/PI3K/AKT signalling pathway as our target. We found that the signalling pathway was inhibited in aspirin group compared with induction only group. Next, we explored if aspirin inhibited the adipogenesis of TSCs through inhibition of PTEN/PI3K/AKT signalling pathway. We added signalling activators IGF‐1 and VO‐Ohpic into the aspirin‐treated TSCs. The results suggested that the two activators reversed adipogenesis inhibition of TSCs induced by aspirin. Many scholars have reported that PI3K/AKT signalling has taken part in adipogenic differentiation process of stem cells. Wang et al^24^ suggested that IGFBP2 promoted adipogenic differentiation of BMSCs through activating JNK and Akt signalling significantly. Song et al^25^ showed that PI3K/Akt/GSK‐3*β*/*β*‐catenin signalling pathway took part in the mechanical stress‐induced changes of osteogenic and adipogenic differentiation in BMSCs. Our findings were in accord with the results above, which told us the important role of PTEN/PI3K/AKT in adipogenesis. This could be new target for reversing adipogenesis and fatty acids formation.

The present study also explored the effect of aspirin on injury tendon healing process. Many reports have stated that NSAIDs promoted healing process through moderating inflammation response of soft tissues such as tendon. COX‐2 selective NSAIDs diminish endogenous resolution responses in systemic inflammation murine models.^26^, ^27^ Lipoxin A4 (LXA_4_), which promotes the resolution of inflammation, is released by low‐dose aspirin.^28^, ^29^ Also, 15‐epi LXA_4_, a kind of lipid mediator for lipid metabolism, has proven efficacious in many chronic inflammatory diseases like pulmonary inflammation^30^ and human infantile eczema.^31^ Dakin et al^32^ showed that 15‐epi LXA_4_ increased the expression of CD206, ALOX15, and CCL22 mRNA and reduced the expression of IL12B mRNA of LPS‐treated diseased tendon‐derived stromal cells compared to no 15‐epi LXA_4_. It is possible that low‐dose aspirin and its metabolites have anti‐inflammation and pro‐resolution effect.^29^, ^32^ Cao et al^33^ showed that BMSC‐promoted calvarial bone defects repair after treated with aspirin in a mini swine model. The results showed that aspirin played a vital role in BMSC‐mediated calvarial bone regeneration. Our previous study revealed that aspirin inhibited inflammation of injury tendon and accelerated healing processes.^18^ The present study showed that aspirin reversed the adipogenic differentiation process, which may be independent of the inflammation regulation and pain relief, and this kind of effect provided a new viewpoint for us to understand that aspirin promoted healing process.

In the current study, the biomechanical properties of injury tendon were increased by aspirin treatment, suggesting that aspirin treatment decreased the risk of injury tendon rupture. Our previous study has proved that aspirin improved the biomechanical properties of injury tendon through regulating inflammation and extracellular matrix formation.^18^ The present study showed that aspirin reversed the fate of TSCs adipogenic differentiation and inhibited lipids accumulation and infiltration in injury tendon; hence, the biomechanical properties were improved. We can imagine that risk of rupture decreased.

There are still several limitations in this study. Firstly, we did not expand the study on patients in clinical, and TSCs were not derived from human beings. Secondly, we did not verify the effect of VO‐Ohpic and IGF‐1 on fatty formation in vivo. Lastly, some long‐term following up should be made in clinical to verify effect of NSAIDs on fatty formation and healing quality of the injury tendon.

In summary, we found that aspirin inhibited adipogenic differentiation of TSCs and fatty infiltration, increased biomechanical properties of injury tendon and promoted healing process of injury tendon. We have demonstrated that PTEN/PI3K/AKT signalling pathway took part in the process above. We suggested that aspirin and its ramifications may contribute to treating tendon injury for their inhibition of fatty infiltration and promotion of biomechanical properties. All these provided new therapeutic potential and medicine evidence of aspirin in treating tendon injury and tendinopathy.

## CONFLICT OF INTEREST

The authors declare that there are no any conflicts of competing financial interests with the contents of this article.

## AUTHOR CONTRIBUTION

YJW participated in the animal experiment, histological experiment, experimental design, acquisition of data, data analysis and interpretation, and manuscript writing. GH acquired the experimental data of the immunostaining, Western blot and qRT‐PCR. FW, CKZ and XLZ joined the experimental design and manuscript revision. HHD and ZLG contributed to the experimental design. CSY, XT and BHZ modified grammar and polished the manuscript. JQZ took part in the conception and design. KLT conducted the conception, design and manuscript writing. All authors read and approved the final manuscript.

## Data Availability

The data supporting the findings of this study are available within the article and its supplementary information files.

## References

[1] Sharma P , Maffulli N . Tendinopathy and tendon injury: the future. Disabil Rehabil. 2008;30:1733‐1745. 18608377 10.1080/09638280701788274

[2] Wu F , Nerlich M , Docheva D . Tendon injuries: basic science and new repair proposals. EFORT Open Rev. 2017;2:332‐342. 28828182 10.1302/2058-5241.2.160075 PMC5549180

[3] Bi YM , Ehirchiou D , Kilts TM , et al. Identification of tendon stem/progenitor cells and the role of the extracellular matrix in their niche. Nat Med. 2007;13:1219‐1227. 17828274 10.1038/nm1630

[4] Rui YF , Lui P , Li G , Fu SC , Lee YW , Chan KM . Isolation and characterization of multipotent rat tendon‐derived stem cells. Tissue Eng Pt A. 2010;16:1549‐1558. 10.1089/ten.TEA.2009.0529 20001227

[5] Zhang J , Wang JH . Characterization of differential properties of rabbit tendon stem cells and tenocytes. BMC Musculoskelet Disord. 2010;11:10. 20082706 10.1186/1471-2474-11-10 PMC2822826

[6] Chen YJ , Wang CJ , Yang KD , et al. Extracorporeal shock waves promote healing of collagenase‐induced Achilles tendinitis and increase TGF‐beta1 and IGF‐I expression. J Orthop Res. 2004;22:854‐861. 15183445 10.1016/j.orthres.2003.10.013

[7] Bez M , Kremen TJ , Tawackoli W , et al. Ultrasound‐mediated gene delivery enhances tendon allograft integration in mini‐pig ligament reconstruction. Mol Ther. 2018;26:1746‐1755. 29784586 10.1016/j.ymthe.2018.04.020 PMC6035740

[8] Sun J , Mou CC , Shi Q , et al. Controlled release of collagen‐binding SDF‐1 alpha from the collagen scaffold promoted tendon regeneration in a rat Achilles tendon defect model. Biomaterials. 2018;162:22‐33. 29428676 10.1016/j.biomaterials.2018.02.008

[9] Antman EM . Evaluating the cardiovascular safety of nonsteroidal anti‐inflammatory drugs. Circulation. 2017;135:2062‐2072. 28533319 10.1161/CIRCULATIONAHA.117.027288

[10] Etminan M , Gill S , Samii A . Effect of non‐steroidal anti‐inflammatory drugs on risk of Alzheimer's disease: systematic review and meta‐analysis of observational studies. BMJ. 2003;327:128. 12869452 10.1136/bmj.327.7407.128 PMC165707

[11] Gray RT , Coleman HG , Hughes C , Murray LJ , Cardwell CR . Low‐dose aspirin use and survival in colorectal cancer: results from a population‐based cohort study. BMC Cancer. 2018;18:1-18. 29486728 10.1186/s12885-018-4142-y PMC6389196

[12] Wang Y , Tang H , He G , et al. High concentration of aspirin induces apoptosis in rat tendon stem cells via inhibition of the Wnt/beta‐catenin pathway. Cell Physiol Biochem: Int J Exp Cell Physiol Biochem Pharmacol. 2018;50:2046‐2059. 10.1159/000495050 30415260

[13] Lui P , Maffulli N , Rolf C , Smith R . What are the validated animal models for tendinopathy? Scand J Med Sci Spor. 2011;21:3‐17. 10.1111/j.1600-0838.2010.01164.x 20673247

[14] Dirks RC , Warden SJ . Models for the study of tendinopathy. J Musculoskelet Neuronal Interact. 2011;11:141‐149. 21625051

[15] Warden SJ . Animal models for the study of tendinopathy. Br J Sports Med. 2007;41:232‐240. 17127722 10.1136/bjsm.2006.032342 PMC2658951

[16] Wang Y , He G , Guo Y , et al. Exosomes from tendon stem cells promote injury tendon healing through balancing synthesis and degradation of the tendon extracellular matrix. J Cell Mol Med. 2019. 10.1111/jcmm.14430 PMC6653097 31148334

[17] Wang Y , Xie L , Tian E , et al. Oncostatin M inhibits differentiation of rat stem Leydig cells in vivo and in vitro. J Cell Mol Med. 2019;23:426‐438. 30320465 10.1111/jcmm.13946 PMC6307848

[18] Wang Y , He G , Tang H , et al. Aspirin inhibits inflammation and scar formation in the injury tendon healing through regulating JNK/STAT‐3 signalling pathway. Cell Prolif. 2019;52:e12650. 31225686 10.1111/cpr.12650 PMC6668964

[19] Ni M , Lui PP , Rui YF , et al. Tendon‐derived stem cells (TDSCs) promote tendon repair in a rat patellar tendon window defect model. J Orthopaedic Res. 2012;30:613‐619. 10.1002/jor.21559 21928428

[20] Heinemeier KM , Ohlenschlaeger TF , Mikkelsen UR , et al. Effects of anti‐inflammatory (NSAID) treatment on human tendinopathic tissue. J Appl Physiol. 1985;2017(123):1397‐1405. 10.1152/japplphysiol.00281.2017 28860166

[21] Yuan M , Zhan Y , Hu W , et al. Aspirin promotes osteogenic differentiation of human dental pulp stem cells. Int J Mol Med. 2018;42:1967‐1976. 30085338 10.3892/ijmm.2018.3801 PMC6108875

[22] Abd Rahman F , Mohd Ali J , Abdullah M , Abu Kasim NH , Musa S . Aspirin enhances osteogenic potential of periodontal ligament stem cells (PDLSCs) and modulates the expression profile of growth factor‐associated genes in PDLSCs. J Periodontol. 2016;87:837‐847. 26846966 10.1902/jop.2016.150610

[23] Zeng YP , Yang C , Li Y , et al. Aspirin inhibits osteoclastogenesis by suppressing the activation of NF‐kappaB and MAPKs in RANKL‐induced RAW264.7 cells. Mol Med Rep. 2016;14:1957‐1962. 27430169 10.3892/mmr.2016.5456 PMC4991763

[24] Wang Y , Liu Y , Fan Z , Liu D , Wang F , Zhou Y . IGFBP2 enhances adipogenic differentiation potentials of mesenchymal stem cells from Wharton's jelly of the umbilical cord via JNK and Akt signaling pathways. PLoS ONE. 2017;12:e0184182. 28859160 10.1371/journal.pone.0184182 PMC5578624

[25] Song F , Jiang D , Wang T , et al. Mechanical stress regulates osteogenesis and adipogenesis of rat mesenchymal stem cells through PI3K/Akt/GSK‐3beta/beta‐catenin signaling pathway. Biomed Res Int. 2017;2017:6027402. 28286769 10.1155/2017/6027402 PMC5329655

[26] Gilroy DW , Lawrence T , Perretti M , Rossi AG . Inflammatory resolution: new opportunities for drug discovery. Nat Rev Drug Discovery. 2004;3:401‐416. 15136788 10.1038/nrd1383

[27] Gilroy DW , Colville‐Nash PR , Willis D , Chivers J , Paul‐Clark MJ , Willoughby DA . Inducible cyclooxygenase may have anti‐inflammatory properties. Nat Med. 1999;5:698‐701. 10371510 10.1038/9550

[28] Serhan CN , Levy BD , Clish CB , Gronert K , Chiang N . Lipoxins, aspirin‐triggered 15‐epi‐lipoxin stable analogs and their receptors in anti‐inflammation: a window for therapeutic opportunity. Ernst Schering Research Foundation workshop.2000;143‐185. 10.1007/978-3-662-04047-8_8 10943332

[29] Morris T , Stables M , Hobbs A , et al. Effects of low‐dose aspirin on acute inflammatory responses in humans. J Immunol. 2009;183:2089‐2096. 19597002 10.4049/jimmunol.0900477

[30] Levy BD , Lukacs NW , Berlin AA , et al. Lipoxin A4 stable analogs reduce allergic airway responses via mechanisms distinct from CysLT1 receptor antagonism. FASEB J. 2007;21:3877‐3884. 17625069 10.1096/fj.07-8653com PMC3005621

[31] Wu SH , Chen XQ , Liu B , Wu HJ , Dong L . Efficacy and safety of 15(R/S)‐methyl‐lipoxin A(4) in topical treatment of infantile eczema. Br J Dermatol. 2013;168:172‐178. 22834636 10.1111/j.1365-2133.2012.11177.x

[32] Dakin SG , Martinez FO , Yapp C , et al. Inflammation activation and resolution in human tendon disease. Sci Transl Med. 2015;7:311ra173. 10.1126/scitranslmed.aac4269 PMC4883654 26511510

[33] Cao Y , Xiong J , Mei S , et al. Aspirin promotes bone marrow mesenchymal stem cell‐based calvarial bone regeneration in mini swine. Stem Cell Res Ther. 2015;6:210. 26519141 10.1186/s13287-015-0200-4 PMC4628405

