# Supplementary figures and images for: Aspirin inhibits adipogenesis of tendon stem cells and lipids accumulation in rat injury tendon through regulating PTEN/PI3K/AKT signalling
Source: J Cell Mol Med. 2019 Sep 26;23(11):7535–44. doi: 10.1111/jcmm.14622 (PMC6815914; doi:10.1111/jcmm.14622)

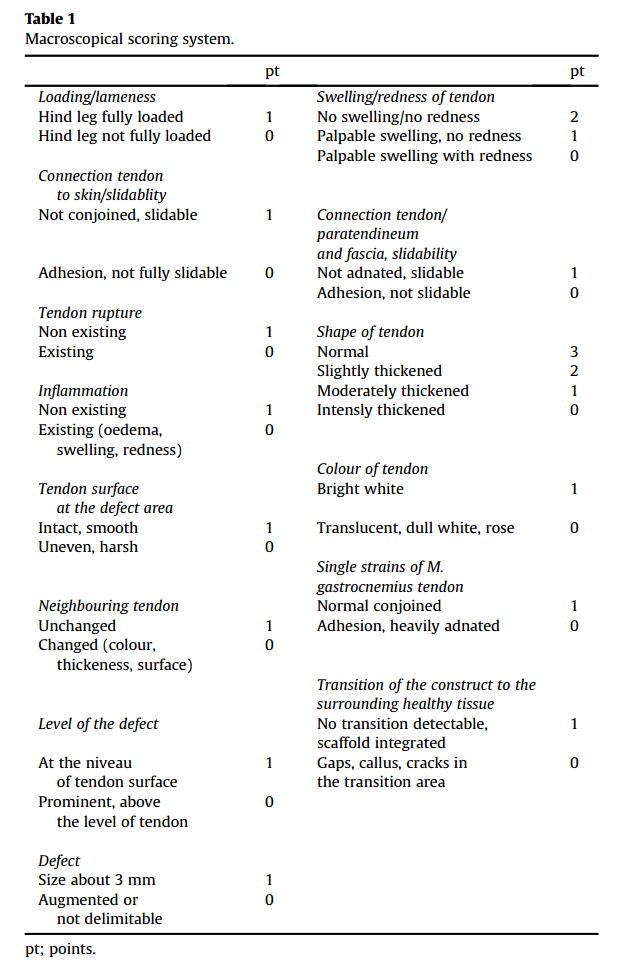


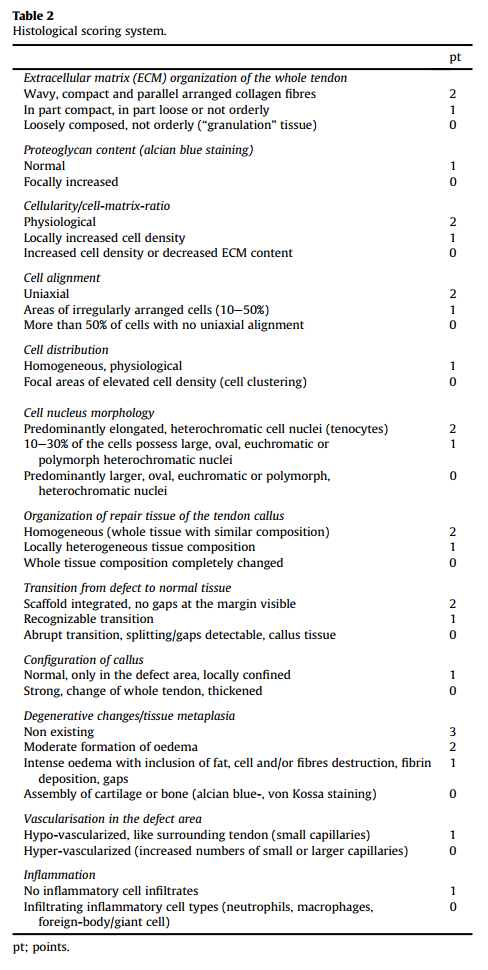

Supplement: Supplementary file 1 [file JCMM-23-7535-s001.docx]
