# Supplementary material for: Aspirin inhibits adipogenesis of tendon stem cells and lipids accumulation in rat injury tendon through regulating PTEN/PI3K/AKT signalling
Source: J Cell Mol Med. 2019 Sep 26;23(11):7535–44. doi: 10.1111/jcmm.14622 (PMC6815914; doi:10.1111/jcmm.14622)
Supplement: Supplementary file 2 [file JCMM-23-7535-s002.docx]

Primer sequences used in the study.

| Gene | Sequences |
| --- | --- |
| ap2 | Forward: CGAGATTTCCTTCAAACTGGG  Reverse: CTTGTAGAAGTCACGCCTTTC |
| PPARγ | Forward: CGGTTGATTTCTCCAGCATTTC  Reverse: CTTCAATCGGATGGTTCTTCGG |
| C/EBPα | Forward: GGTGGATAAGAACAGCAACG  Reverse: GGTCATTGTCACTGGTCAAC |
| GAPDH | Forward: AGAAGGCTGGGGCTCATTTG  Reverse: AGGGGCCATCCACAGTCTTC |
